# Clinical mechanisms of repetitive transcranial magnetic stimulation in improving constipation in Parkinson’s disease patients through the gut-brain axis

**DOI:** 10.3389/fnagi.2025.1607791

**Published:** 2025-08-05

**Authors:** Qianlan Bo, Yanmin Li, Xiayue Wang, Huijun Wang, Huimiao Liu

**Affiliations:** ^1^Department of Neurology, The First Hospital of Hebei Medical University, Shijiazhuang, Hebei, China; ^2^Brain Aging and Cognitive Neuroscience Laboratory of Hebei Province, Shijiazhuang, Hebei, China; ^3^Neuromedical Technology Innovation Center of Hebei Province, Shijiazhuang, Hebei, China

**Keywords:** repetitive transcranial magnetic stimulation, gut-brain axis, Parkinson’s disease, constipation, non-motor symptoms, neuromodulation

## Abstract

**Objectives:**

This study investigates the clinical efficacy of repetitive transcranial magnetic stimulation (rTMS) in alleviating constipation in patients with Parkinson’s disease (PD) via the gut-brain axis.

**Methods:**

Fifty-eight PD patients with constipation, admitted from May 2023 to December 2024, were randomly assigned to an rTMS treatment group or a sham rTMS control group (29 patients each). Chronic constipation severity was assessed using the Chronic Constipation Severity Score (CSS) before and 14 days after treatment. Additional measures included weekly spontaneous bowel movements (SBM), complete spontaneous bowel movements (CSBM), the Bristol Stool Scale (BSS), and serum levels of gut-brain peptides (5-HT, BDNF, VIP) and cytokines (IL-6, IFN-γ, TNF-α, IL-4, IL-10).

**Results:**

Baseline characteristics, including CSS scores, were similar between groups (*P* > 0.05). After 14 days, the study group exhibited significantly lower CSS scores compared to the control group (e.g., CSS post-treatment: study group 4.03 ± 1.01 vs. control group 6.23 ± 1.03, *P* < 0.001). Both groups showed increased SBM and CSBM frequencies; however, the study group demonstrated significantly higher counts (e.g., CSBM post-treatment: study group 4.67 ± 0.04 vs. control group 4.16 ± 0.06, *P* < 0.001). BSS scores improved in both groups, with the study group achieving significantly higher scores (*P* < 0.05). Post-treatment, the study group had significantly higher serum 5-HT (*P* < 0.001) and BDNF (*P* = 0.001) levels, and lower VIP levels (*P* = 0.041) compared to the control group. Cytokine analysis revealed significantly lower pro-inflammatory IL-6 (*P* < 0.001), IFN-γ (*P* = 0.034), TNF-α (*P* < 0.001) and higher anti-inflammatory IL-4 (*P* < 0.001), IL-10 (*P* < 0.001) levels in the study group, with corresponding Cohen’s d values indicating medium to very large effect sizes.

**Conclusion:**

Repetitive transcranial magnetic stimulation effectively improves constipation symptoms in PD patients over a 14 days period. These benefits are associated with favorable modulations of gut-brain peptides and cytokine profiles, suggesting a therapeutic mechanism involving the gut-brain axis. However, direct causality and the long-term effects require further investigation.

## 1 Introduction

Parkinson’s disease (PD) is primarily known for its characteristic motor symptoms, but it is also accompanied by a range of non-motor symptoms (NMS), which can significantly affect the quality of life of patients. One of the most prevalent and debilitating NMS in PD patients is constipation, affecting a significant proportion of this population, with reported incidences ranging from 27.10% to 70.39% (Diaconu and Falup-Pecurariu, 2022; Henderson, 2022). Constipation often manifests before motor symptoms and tends to worsen with disease progression, severely impacting daily functioning and potentially influencing levodopa absorption, thereby contributing to motor fluctuations in advanced PD (Diaconu and Falup-Pecurariu, 2022; Henderson, 2022).

The exact mechanism underlying constipation in PD remains unclear, though it is considered multifactorial. Key pathophysiological processes include degeneration of central nervous system pathways and α-synuclein deposition within the enteric nervous system (ENS) (Elangovan et al., 2024; Zhao et al., 2021). These changes can lead to decreased gut motility, increased colonic transit time, and impaired rectal contractility. Recent research emphasizes the “gut-brain axis,” a bidirectional communication system between the gastrointestinal tract and the central nervous system (CNS), as crucial in PD pathogenesis and its associated constipation (Elangovan et al., 2024; Zhao et al., 2021). This axis involves neural (e.g., vagus nerve), endocrine (e.g., gut hormones), and immune pathways. Dysregulation of gut-brain peptides such as serotonin (5-HT), brain-derived neurotrophic factor (BDNF), and vasoactive intestinal peptide (VIP) is implicated in the impaired gut function seen in PD (Elangovan et al., 2024).

Repetitive transcranial magnetic stimulation (rTMS) is a non-invasive neuromodulation technique that has shown promise in treating various symptoms of PD (Lai et al., 2023). By modulating cortical excitability, rTMS can influence subcortical structures and neurotransmitter systems, including autonomic pathways that connect to the gastrointestinal tract. Given this connection, rTMS targeted at specific cortical regions might exert downstream effects on the gut-brain axis. However, the impact of rTMS on PD-related constipation remains underexplored. We hypothesize that rTMS, by modulating central neural circuits connected to the ENS, could influence the secretion of gut-brain peptides and cytokines involved in gut motility and inflammation, thereby improving gastrointestinal function and alleviating constipation in PD patients.

This study aims to examine the effects of rTMS treatment on constipation symptoms in Parkinson’s disease by measuring constipation-related clinical scores and serum levels of key gut-brain peptides (5-HT, BDNF, VIP) and a panel of pro-inflammatory (IL-6, IFN-γ, TNF-α) and anti-inflammatory (IL-4, IL-10) cytokines before and after a 14 days intervention. The findings are expected to provide theoretical support for rTMS efficacy and contribute to a deeper understanding of its mechanisms in alleviating constipation in PD via the gut-brain axis, potentially offering new therapeutic avenues.

## 2 Materials and methods

### 2.1 Clinical data

A total of 66 patients with Parkinson’s disease (PD) and constipation were initially screened for enrollment at our hospital between May 2023 and December 2023. Of these, eight patients were excluded prior to randomization (three due to gastric bleeding, three with gastric ulcers, and two with cardiac pacemakers). Consequently, 58 eligible patients were included in this study. The study protocol complies with the relevant requirements of the Declaration of Helsinki issued by the World Medical Association and has been approved by the ethics committee of The First Hospital of Hebei Medical University (2024-178). All participants provided written informed consent.

### 2.2 Inclusion, exclusion, and withdrawal criteria

#### 2.2.1 Inclusion criteria

(1) Diagnosed with Parkinson’s disease according to the 2015 Movement Disorder Society (MDS) clinical diagnostic criteria for Parkinson’s disease (Postuma et al., 2015);
(2) Diagnosed with functional constipation based on the Rome III diagnostic criteria (Chouliaras et al., 2021);
(3) Age under 80 years, regardless of gender;
(4) Able to understand and cooperate in completing all examination procedures;
(5) Signed informed consent.

#### 2.2.2 Exclusion criteria

(1) Patients previously diagnosed with Parkinson’s syndrome (including traumatic, tumor-related, vascular, drug-induced, toxic, or hydrocephalus-related) or those with severe cardiovascular, cerebral, renal, or other systemic diseases;
(2) Patients with intracranial organic diseases or epilepsy;
(3) Patients with implanted cardiac pacemakers or other metal implants;
(4) Patients with a history of active gastrointestinal diseases (e.g., gastrointestinal tumors, current bleeding, active ulcers);
(5) Patients unable to complete scale assessments.
(6) Contraindications to rTMS.

#### 2.2.3 Withdrawal criteria

(1) Subjects experience severe changes in their condition during the trial, such as significant organ dysfunction, intolerance to treatment, poor compliance, disease worsening, or severe adverse reactions;
(2) The investigator decides to withdraw the subject from the study;
(3) The subject feels the treatment is ineffective, cannot tolerate side effects, wishes to adopt other treatments, or voluntarily requests to withdraw for any reason.

### 2.3 Treatment methods

#### 2.3.1 Conventional western medication

All patients received routine treatment for PD based on the 2020 “Chinese Parkinson’s Disease Treatment Guidelines (4th edition).” Basic treatment involved using Madopar (Levodopa + Benserazide, Shanghai Roche Pharmaceuticals Co., Ltd., 0.25 g/tablet, National Drug Standard H10930198). The optimal treatment plan was selected based on individual patient condition under specialist guidance, and the effective maintenance dose was administered. For constipation, all patients received lactulose oral solution (Manufacturer: Abbott Healthcare Products B.V., approval number: H20171057) once daily, 30 ml before breakfast, as a standard background treatment. Throughout the 14 days study period, dosages of PD medications and lactulose were kept stable for all participants. Patients were also instructed to maintain their usual dietary habits and physical activity levels to minimize these potential confounding factors.

#### 2.3.2 Treatment group

On the basis of conventional treatment, patients were treated using the MagproR 30 magnetic stimulation device (Medtronic, Denmark). Patients were positioned comfortably, either lying down or sitting with eyes closed. An “8”-shaped coil (unilateral outer diameter 70 mm) was used. The resting motor threshold (MT) was determined for each participant by identifying the lowest stimulator output intensity that elicited a visible twitch in the contralateral abductor pollicis brevis (APB) muscle in at least 5 out of 10 trials when stimulating the primary motor cortex hand area. The peak magnetic field strength was set at 4.2 T, with a stimulation frequency of 5 Hz and an intensity of 100% of the motor threshold, delivering 1,800 pulses. These parameters were chosen based on established protocols for rTMS in neurological disorders, particularly for stimulating the dorsolateral prefrontal cortex (DLPFC). A frequency of 5 Hz is considered high-frequency stimulation, known to enhance cortical excitability, and has been used in studies for PD and related non-motor symptoms. Stimulation at 100% of the motor threshold is a common intensity ensuring effective cortical stimulation while maintaining safety. The total number of pulses (1,800) per session is within the range reported in studies demonstrating therapeutic effects and adheres to safety guidelines (Lefaucheur et al., 2020). The position of the stimulation coil was adjusted to ensure it was tangential to the patient’s scalp, targeting the left dorsolateral prefrontal cortex for 20 min per session, once daily.

#### 2.3.3 Control group

In addition to their existing PD medication and lactulose, patients received sham rTMS. The same “8”-shaped coil was used, positioned over the left DLPFC with the same visual and auditory cues as active rTMS (e.g., clicking sound from the machine). However, the coil was tilted at a 90-degree angle relative to the scalp, ensuring that the magnetic field did not significantly penetrate the cortex, a commonly accepted method for sham stimulation designed to maintain blinding (Lefaucheur et al., 2020). The duration was 20 min per session, once daily.

#### 2.3.4 Treatment duration

Both groups underwent treatment for a duration of 14 days.

### 2.4 Observation indicators

The following parameters were observed 1 h before the first stimulation session and 14 days after the last treatment session:

• Chronic Constipation Severity Score (CSS) (Higashimori et al., 2021)
• Frequency of spontaneous bowel movements (SBM) per week
• Frequency of complete spontaneous bowel movements (CSBM) per week
• Bristol Stool Scale (BSS) score (Lai et al., 2023)
• Serum levels of gut-brain peptides including serotonin (5-HT), brain-derived neurotrophic factor (BDNF), vasoactive intestinal peptide (VIP).
• Serum levels of cytokines: Interleukin-6 (IL-6), Interferon-gamma (IFN-γ), Tumor Necrosis Factor-alpha (TNF-α), Interleukin-4 (IL-4), and Interleukin-10 (IL-10).

#### 2.4.1 Chronic Constipation Severity Score (CSS)

A questionnaire was used to assess the patient’s constipation symptoms (Higashimori et al., 2021). The patient rated the severity of symptoms, including difficulty defecating, defecation frequency, sensation of incomplete evacuation, abdominal pain, time spent per bowel movement, use of laxatives, failed attempts at defecation, and the duration of constipation. Scores ranged from 0 to 4 for each symptom, with higher scores indicating more severe constipation.

#### 2.4.2 Bristol Stool Scale (BSS)

The stool consistency was scored as follows (Lai et al., 2023):

• Type 1: Separate hard lumps, like nuts (difficult to pass) – score 1
• Type 2: Sausage-shaped but lumpy – score 2
• Type 3: Like a sausage but with cracks on the surface – score 3
• Type 4: Like a sausage or snake, smooth and soft – score 4
• Type 5: Soft blobs with clear-cut edges (passed easily) – score 5
• Type 6: Fluffy pieces with ragged edges, a mushy stool – score 6
• Type 7: Watery, no solid pieces – score 7

#### 2.4.3 Laboratory indicators

Venous blood (3 ml) was collected 1 h before the first stimulation and 14 days after the last stimulation. Samples were centrifuged at 3,000 rpm for 20 min. Serum levels of 5-HT, BDNF, VIP, IL-6, IFN-γ, TNF-α, IL-4, and IL-10 were measured using enzyme-linked immunosorbent assays (ELISA) kits according to manufacturer instructions. Units were standardized: 5-HT (pg/mL), BDNF (pg/mL), and VIP (pg/mL).

### 2.5 Statistical analysis

Data analysis was performed using SPSS 26.0 software. Normality of continuous data was assessed using the Shapiro-Wilk test. Continuous data conforming to a normal distribution were expressed as mean ± standard deviation (x̄ ± s) and compared between two independent groups using the independent samples *t*-test. Data not conforming to normal distribution would be analyzed using the Mann-Whitney U test, though all primary continuous outcomes in this study were found to be normally distributed. Categorical data were expressed as frequencies (percentages) and compared using the chi-square test (χ^2^) or Fisher’s exact test when expected cell counts were less than 5. Intragroup comparisons (pre- vs. post-treatment) were made using paired *t*-tests. Effect sizes (Cohen’s d) were calculated for significant intergroup differences in all primary and secondary outcomes to quantify the magnitude of the rTMS impact. A Pearson correlation analysis was performed to explore the relationship between changes in clinical scores (e.g., ΔCSS) and changes in biomarker levels within the treatment group. Repeated-measures ANOVA was also performed as a supplementary analysis to confirm significant group × time interactions (see Supplementary Table 1). A *P*-value ≤ 0.05 was considered statistically significant.

## 3 Results

### 3.1 Comparison of general data between the two groups

Fifty-eight PD patients with constipation completed the study and were included in the final analysis (29 in the study group, 29 in the control group). There were no statistically significant differences between the two groups at baseline in terms of age, gender, BMI, alcohol consumption, smoking history, or prevalence of comorbidities such as hypertension, hyperlipidemia, and diabetes (all *P* > 0.05) (Table 1).

**TABLE 1:** Comparison of general data between the two groups.

Item	Study group (*n* = 29)	Control group (*n* = 29)	χ^2^/t	*P*
Gender (*n*)			0.069	0.793
Male	16	15		
Female	13	14		
Age (years)	51.28 ± 7.27	50.98 ± 7.09	−0.159	0.874
PD duration (years)	4.6 ± 2.2	4.9 ± 2.4	0.512	0.611
BMI (kg/m^2^)	23.21 ± 2.56	23.17 ± 2.45	−0.061	0.952
Baseline CSS score	16.47 ± 2.39	16.44 ± 2.32	−0.049	0.962
Alcohol consumption (*n*)	6	9	0.809	0.368
Smoking (*n*)	7	8	0.090	0.764
Hypertension (*n*)	4	6	0.293	0.588^F^
Hyperlipidemia (*n*)	3	4	0.162	0.687^F^
Diabetes (*n*)	3	2	0.219	0.640^F^

^F^*P*-value obtained using Fisher’s exact test due to small expected cell counts.

### 3.2 Comparison of CSS scores between the two groups

**FIGURE 1:**
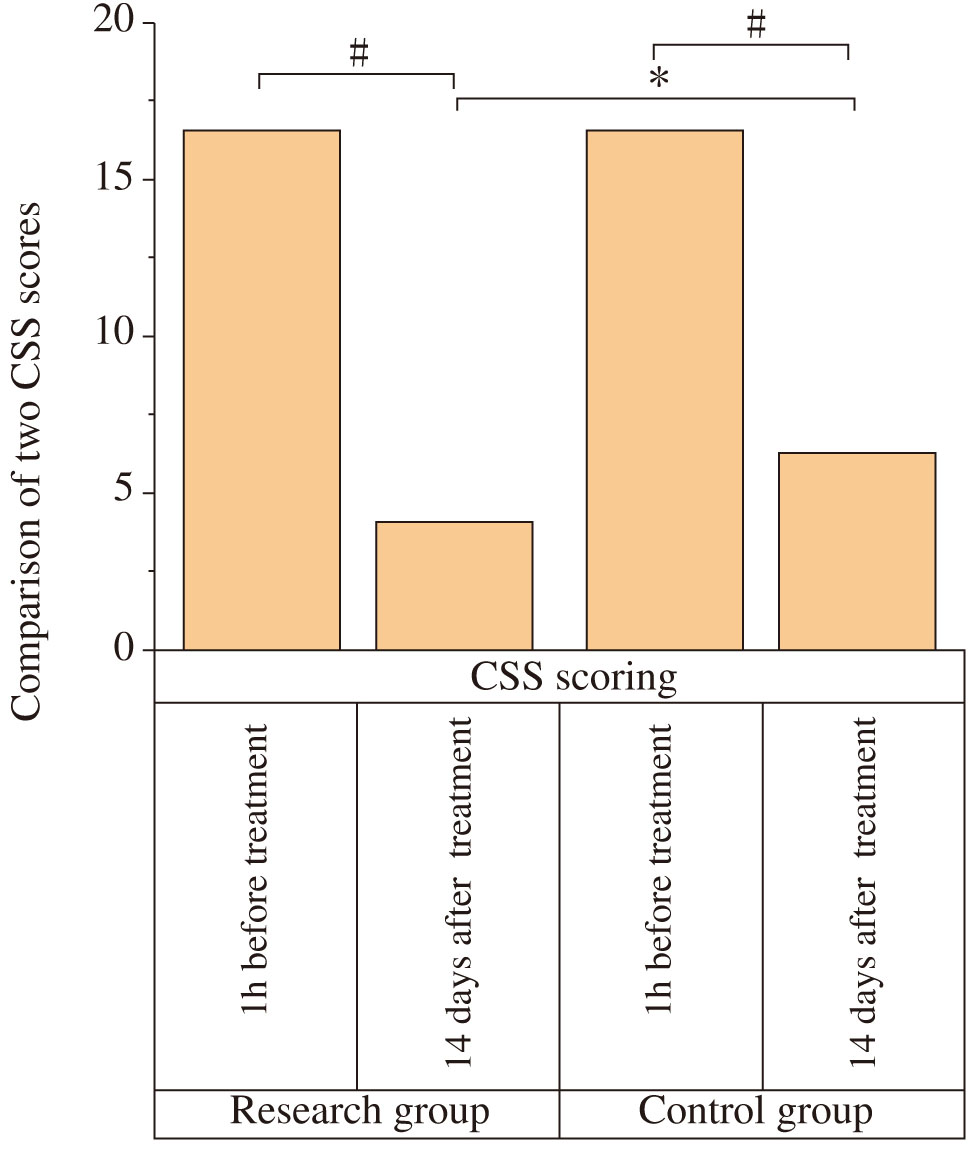
Comparison of Chronic Constipation Severity (CSS) scores between the two groups. The CSS scores were compared between the study and control groups at baseline (1 h before treatment) and after 14 days of treatment. At baseline, there was no significant difference between the two groups (*P* > 0.05). After 14 days of treatment, the CSS scores decreased significantly in both groups compared to baseline (#P < 0.05). The study group showed a significantly lower CSS score than the control group post-treatment (*P < 0.05).

At baseline (1 h before treatment), there was no statistically significant difference in CSS scores between the two groups (*P* > 0.05). After 14 days of treatment, the CSS scores in both groups decreased compared to baseline, and the study group had a lower CSS score than the control group, with a statistically significant difference (*P* < 0.001) and a very large effect size (Cohen’s d = 2.16) (Figure 1 and Table 2). In terms of clinically meaningful improvement, 25 of 29 patients (86.2%) in the study group and 18 of 29 patients (62.1%) in the control group achieved a reduction of ≥ 3 points on the CSS, with the difference being statistically significant (χ^2^ = 4.885, *P* = 0.027).

**TABLE 2:** Comparison of Chronic Constipation Severity (CSS) scores between the two groups.

Time	Study group (*n* = 29)	Control group (*n* = 29)	t	*P*
Pre-treatment (1 h)	16.47 ± 2.39	16.44 ± 2.32	−0.049	0.962
Post-treatment (14 days)	4.03 ± 1.01^#^	6.23 ± 1.03^#^*	8.213	< 0.001

^#^*P* < 0.05 compared with pre-treatment within the same group.

*P < 0.05 compared with the study group at the same time point.

### 3.3 Comparison of SBM and CSBM frequency between the two groups

**FIGURE 2:**
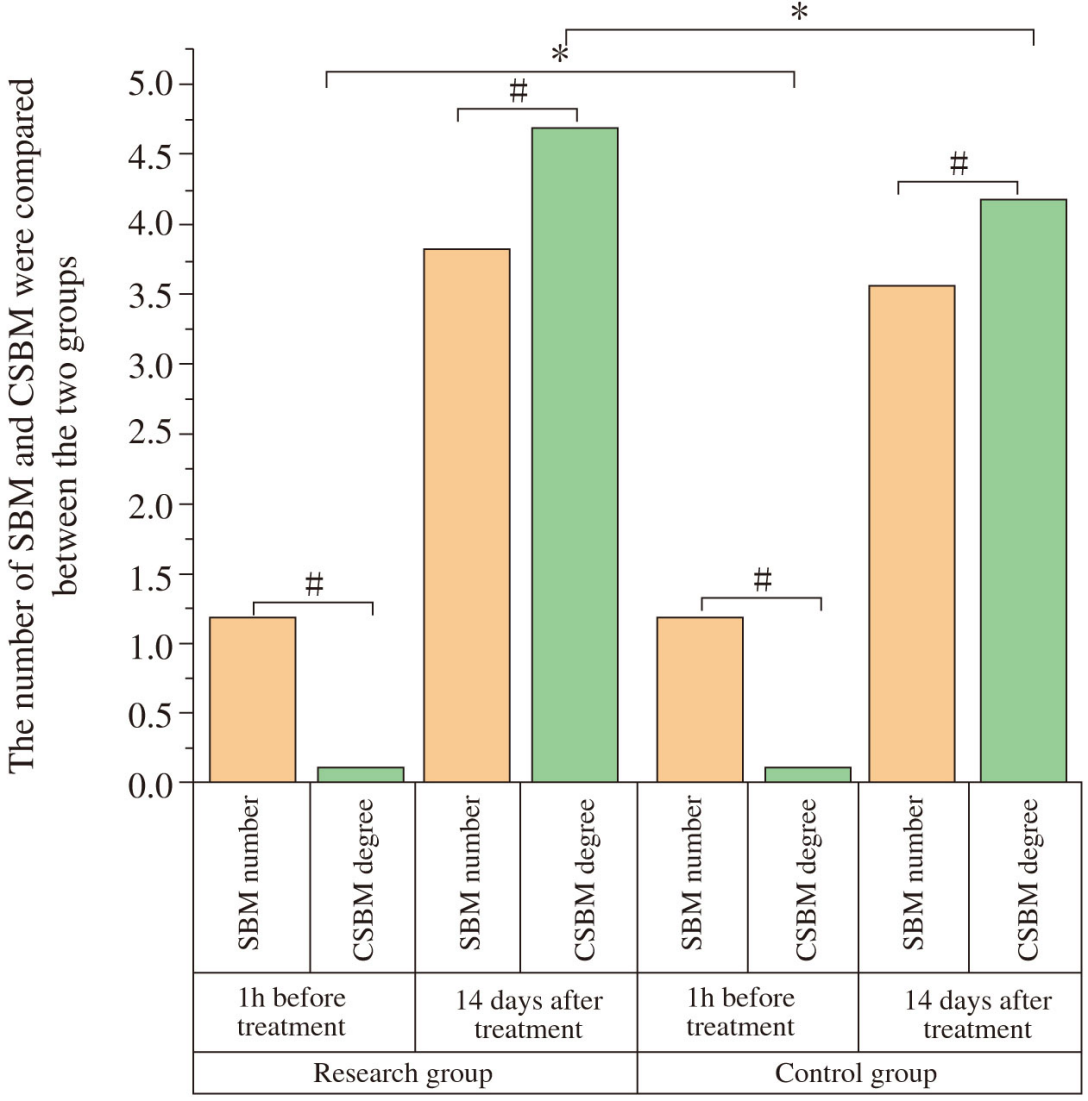
Comparison of spontaneous bowel movements (SBM) and complete spontaneous bowel movements (CSBM) frequency between the two groups. The frequency of SBM and CSBM were compared between the study and control groups at baseline (1 h before treatment) and after 14 days of treatment. At baseline, there was no significant difference in SBM or CSBM frequency between the two groups (*P* > 0.05). After 14 days of treatment, both groups showed a significant increase in SBM and CSBM frequency compared to baseline (#*P* < 0.05). The study group demonstrated significantly higher SBM and CSBM frequencies than the control group post-treatment (*P < 0.05).

At baseline, there were no significant differences in SBM frequency (Study: 1.17 ± 0.18; Control: 1.19 ± 0.16; t = 0.447, *P* = 0.656) or CSBM frequency (Study: 0.11 ± 0.02; Control: 0.10 ± 0.02; t = −1.904, *P* = 0.062) between the groups. After 14 days, both groups showed significant increases in SBM and CSBM frequencies compared to baseline (*P* < 0.05). The study group had significantly higher post-treatment SBM frequency (3.83 ± 0.15) than the control group (3.57 ± 0.17) (t = −6.176, *P* < 0.001; Cohen’s d = 1.63), and significantly higher post-treatment CSBM frequency (4.67 ± 0.04) than the control group (4.16 ± 0.06) (t = −38.086, *P* < 0.001; Cohen’s d = 10.1) (Figure 2 and Table 3). The notably small standard deviation (0.04) for post-treatment CSBM frequency in the study group suggests a highly consistent response among these participants.

**TABLE 3:** Comparison of spontaneous bowel movements (SBM) and complete spontaneous bowel movements (CSBM) frequency between the two groups.

Item	Time	Study group (*n* = 29)	Control group (*n* = 29)	t	*P*
SBM frequency	Pre-treatment (1 h)	1.17 ± 0.18	1.19 ± 0.16	0.447	0.656
	Post-treatment (14 days)	3.83 ± 0.15^#^	3.57 ± 0.17^#^*	−6.176	< 0.001
CSBM frequency	Pre-treatment (1 h)	0.11 ± 0.02	0.10 ± 0.02	−1.904	0.062
	Post-treatment (14 days)	4.67 ± 0.04^#^	4.16 ± 0.06^#^*	−38.086	< 0.001

^#^*P* < 0.05 compared with pre-treatment within the same group.

*P < 0.05 compared with the study group at the same time point.

### 3.4 Comparison of BSS scores between the two groups

**FIGURE 3:**
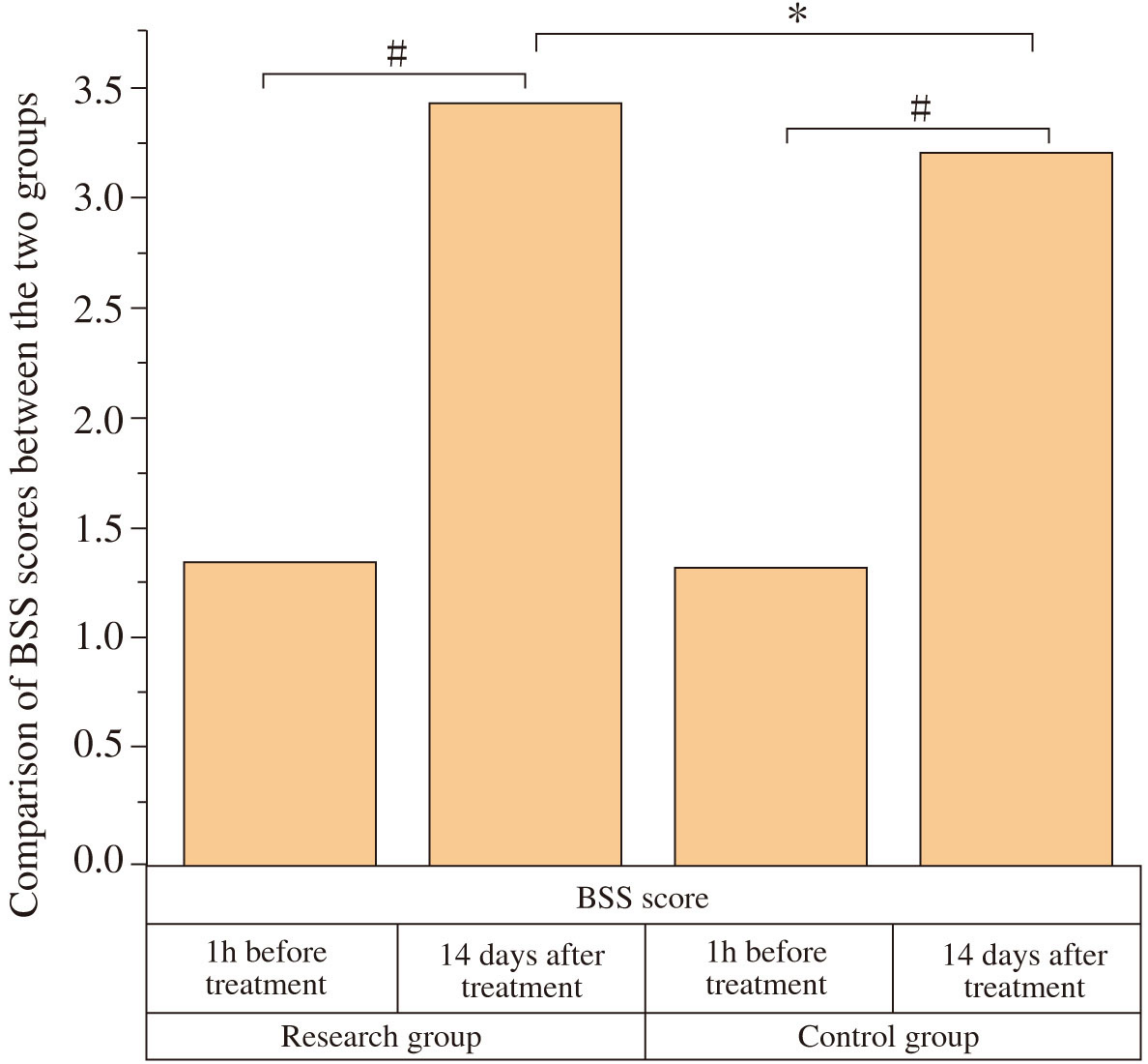
Comparison of Bristol Stool Scale (BSS) scores between the two groups. The BSS scores were compared between the study and control groups at baseline (1 h before treatment) and after 14 days of treatment. At baseline, there was no significant difference in BSS scores between the two groups (*P* > 0.05). After 14 days of treatment, both groups showed a significant improvement in BSS scores compared to baseline (#*P* < 0.05). The study group showed a significantly higher BSS score than the control group post-treatment (*P < 0.05).

At baseline (1 h before treatment), there was no significant difference in BSS scores between the two groups (*P* > 0.05). After 14 days of treatment, both groups showed an improvement in BSS scores compared to baseline, and the study group had a higher BSS score than the control group, with a statistically significant difference (*P* < 0.05, Cohen’s d = 0.67) (Figure 3 and Table 4).

**TABLE 4:** Comparison of Bristol Stool Scale (BSS) scores between the two groups.

Time	Study group (*n* = 29)	Control group (*n* = 29)	t	*P*
Pre-treatment (1 h)	1.37 ± 0.23	1.34 ± 0.21	−0.519	0.606
Post-treatment (14 days)	3.42 ± 0.39^#^	3.19 ± 0.33^#^*	−2.424	0.019

^#^*P* < 0.05 compared with pre-treatment within the same group.

*P < 0.05 compared with the study group at the same time point.

### 3.5 Comparison of serum gut-brain peptide levels between the two groups

**FIGURE 4:**
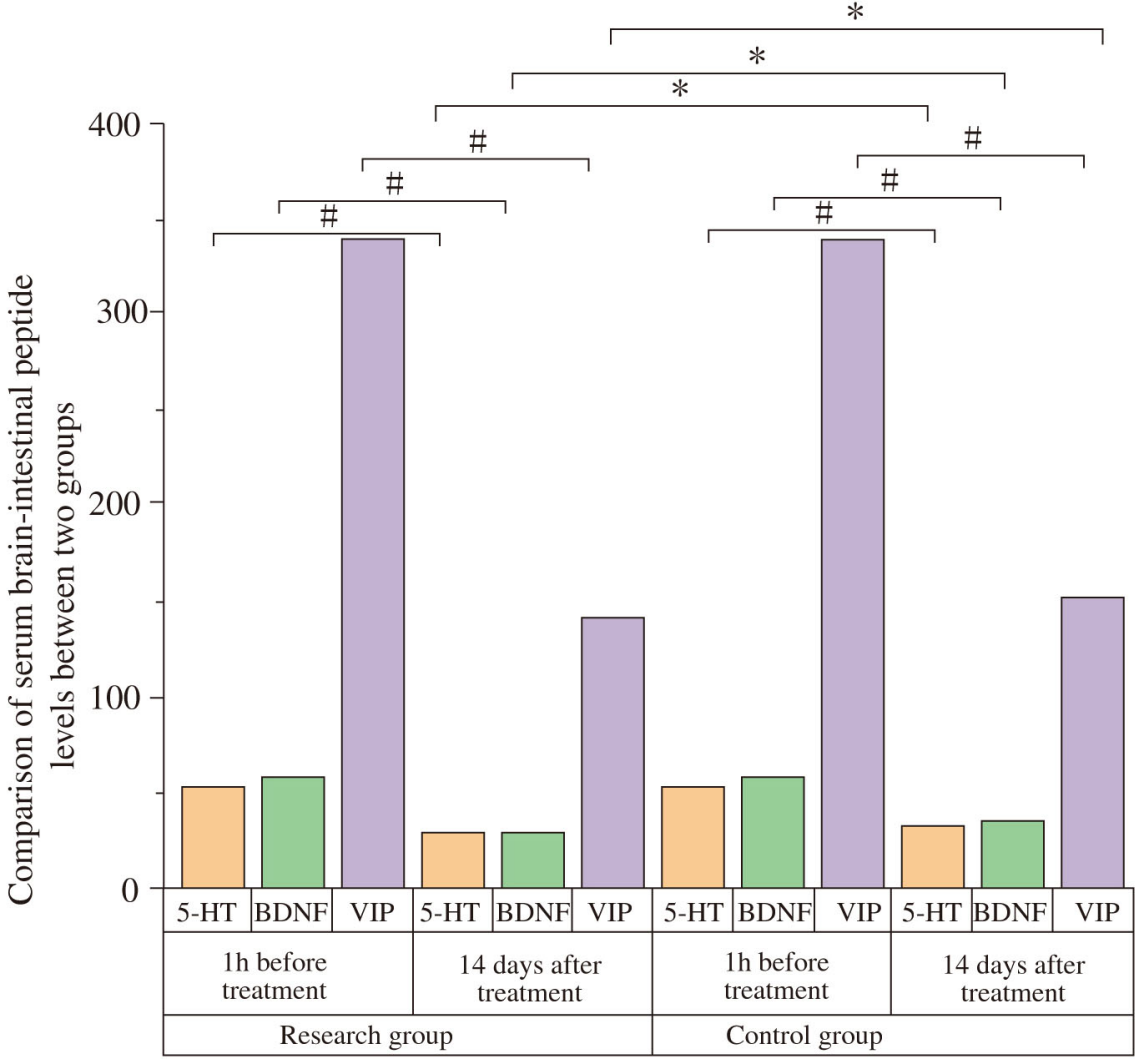
Comparison of serum gut-brain peptides between the two groups. Serum levels of serotonin (5-HT), brain-derived neurotrophic factor (BDNF), and vasoactive intestinal peptide (VIP) were compared between the study and control groups at baseline (1 h before treatment) and after 14 days of treatment. At baseline, there were no significant differences between the groups (*P* > 0.05). After 14 days, 5-HT and BDNF levels significantly increased, while VIP levels significantly decreased in both groups compared to baseline (#*P* < 0.05). Post-treatment, the study group showed significantly higher 5-HT and BDNF levels, and significantly lower VIP levels compared to the control group (*P < 0.05).

One hour before treatment, there were no statistically significant differences in serum levels of 5-HT (Study: 23210 ± 4530 pg/mL; Control: 23170 ± 4490 pg/mL; t = −0.034, *P* = 0.973), BDNF (Study: 29.04 ± 5.39 pg/mL; Control: 28.97 ± 5.63 pg/mL; t = −0.048, *P* = 0.962), or VIP (Study: 339.78 ± 41.29 pg/mL; Control: 338.09 ± 40.76 pg/mL; t = −0.157, *P* = 0.876) between the groups. Fourteen days after treatment, serum 5-HT and BDNF levels significantly increased, while VIP levels significantly decreased in both groups compared to baseline (all *P* < 0.05). Post-treatment, the study group showed significantly higher 5-HT levels (69760 ± 4760 pg/mL) than the control group (53190 ± 4230 pg/mL) (t = −14.013, *P* < 0.001; Cohen’s d = 3.69). The study group also had significantly higher BDNF levels (60.06 ± 5.37 pg/mL) compared to the control group (55.19 ± 5.38 pg/mL) (t = 3.634, *P* = 0.001; Cohen’s d = 0.96). Conversely, the study group had significantly lower VIP levels (140.29 ± 28.27 pg/mL) than the control group (155.24 ± 26.07 pg/mL) (t = 2.094, *P* = 0.041; Cohen’s d = 0.55) (Figure 4 and Table 5).

**TABLE 5:** Comparison of serum gut-brain peptide levels between the two groups.

Peptide (unit)	Time	Study group (*n* = 29)	Control group (*n* = 29)	t	*P*
5-HT (pg/mL)	Pre-treatment (1 h)	23210 ± 4530	23170 ± 4490	−0.034	0.973
	Post-treatment (14 days)	69760 ± 4760^#^	53190 ± 4230^#^*	−14.013	< 0.001
BDNF (pg/mL)	Pre-treatment (1 h)	29.04 ± 5.39	28.97 ± 5.63	−0.048	0.962
	Post-treatment (14 days)	60.06 ± 5.37^#^	55.19 ± 5.38^#^*	3.634	0.001
VIP (pg/mL)	Pre-treatment (1 h)	339.78 ± 41.29	338.09 ± 40.76	−0.157	0.876
	Post-treatment (14 days)	140.29 ± 28.27^#^	155.24 ± 26.07^#^*	2.094	0.041

^#^*P* < 0.05 compared with pre-treatment within the same group.

*P < 0.05 compared with the study group at the same time point.

### 3.6 Comparison of cytokine levels between the two groups

**FIGURE 5:**
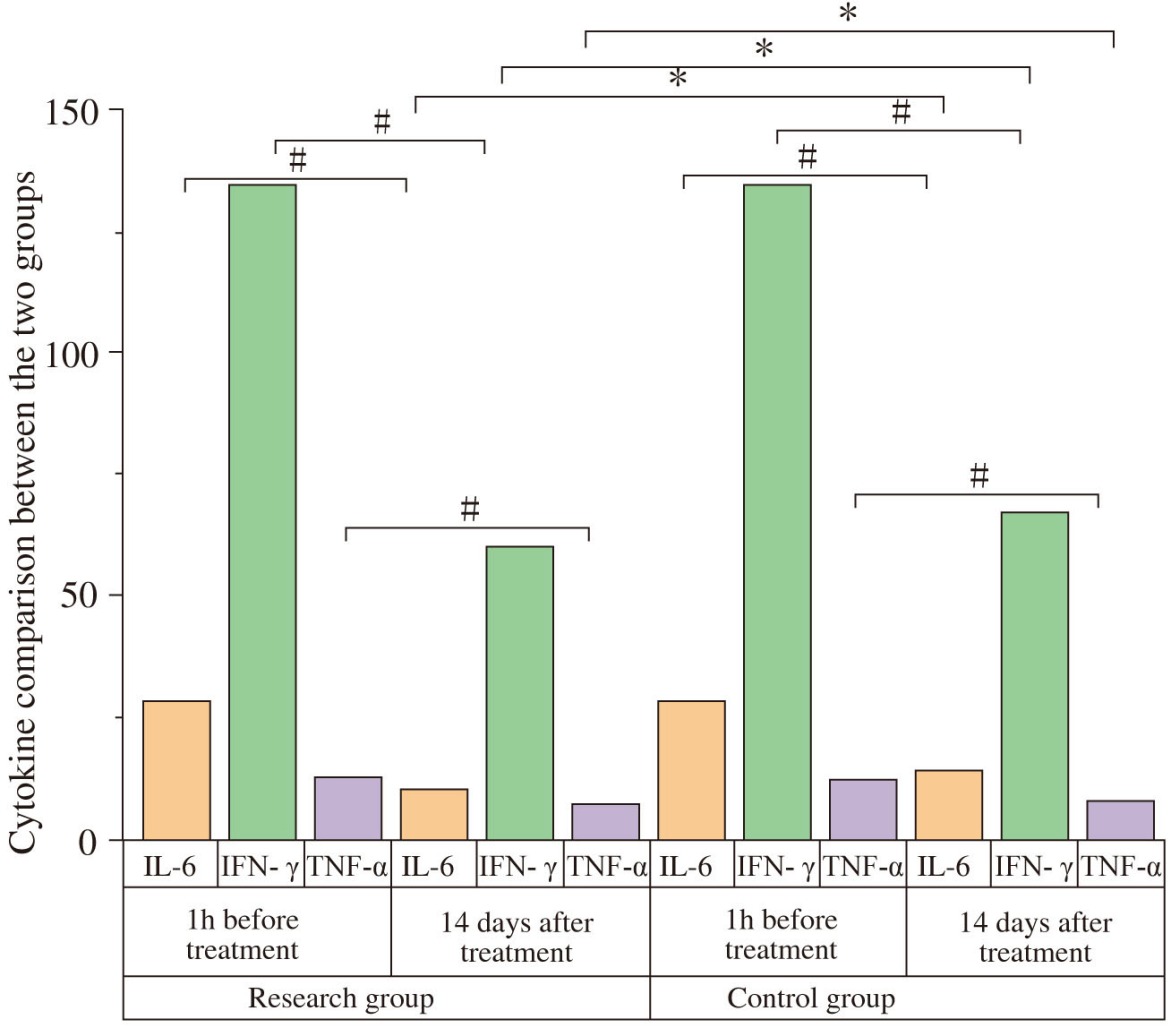
Comparison of cytokine levels between the two groups. Serum levels of IL-6, IFN-γ, TNF-α, IL-4, and IL-10 were compared between the study and control groups at baseline (1 h before treatment) and after 14 days of treatment. At baseline, there were no significant differences between the groups (*P* > 0.05). After 14 days, IL-6, IFN-γ, and TNF-α levels significantly decreased, while IL-4 and IL-10 levels significantly increased in both groups compared to baseline (#*P* < 0.05). Post-treatment, the study group showed significantly lower levels of IL-6, IFN-γ, and TNF-α, and significantly higher levels of IL-4 and IL-10 compared to the control group (*P < 0.05).

At baseline, no significant intergroup differences were found for IL-6, IFN-γ, TNF-α, IL-4, or IL-10 levels (all *P* > 0.05). After 14 days, pro-inflammatory cytokines IL-6, IFN-γ, and TNF-α significantly decreased, while anti-inflammatory cytokines IL-4 and IL-10 significantly increased in both groups compared to baseline (all *P* < 0.05). Post-treatment, the study group exhibited significantly lower levels of IL-6 (Study: 10.23 ± 2. pg/mL; Control: 14.34 ± 2.34 pg/mL; t = 6.409, *P* < 0.001, Cohen’s d = 1.69), IFN-γ (Study: 0.64 ± 0.13 pg/mL; Control: 0.72 ± 0.15 pg/mL; t = 2.170, *P* = 0.034, Cohen’s d = 0.57), and TNF-α (Study: 6.87 ± 1.02 pg/mL; Control: 7.65 ± 1.13 pg/mL; t = 2.759, *P* < 0.001, Cohen’s d = 0.73) compared to the control group. Furthermore, the study group had significantly higher levels of IL-4 (Study: 3.82 ± 0.26 pg/mL; Control: 3.21 ± 0.29 pg/mL; t = −8.434, *P* < 0.001, Cohen’s d = 2.22) and IL-10 (Study: 4.97 ± 0.24 pg/mL; Control: 4.16 ± 0.25 pg/mL; t = −12.587, *P* < 0.001, Cohen’s d = 3.31) than the control group (Figure 5 and Table 6). The negative t-values for IL-4 and IL-10 reflect the order of subtraction in the *t*-test calculation (Control - Study) but confirm that the study group had significantly higher mean values.

**TABLE 6:** Comparison of cytokines between two groups.

Cytokine	Time	Study group (*n* = 29)	Control group (*n* = 29)	t	*P*
IL-6 (pg/mL)	Pre-treatment (1 h)	28.34 ± 2.39	28.29 ± 2.41	−0.079	0.937
	Post-treatment (14 days)	10.23 ± 2.54^#^	14.34 ± 2.34^#^*	6.409	< 0.001
IFN-γ (pg/mL)	Pre-treatment (1 h)	1.29 ± 0.14	1.23 ± 0.12	−1.752	0.085
	Post-treatment (14 days)	0.64 ± 0.13^#^	0.72 ± 0.15^#^*	2.170	0.034
TNF-α (pg/mL)	Pre-treatment (1 h)	12.27 ± 1.34	12.21 ± 1.31	−0.172	0.864
	Post-treatment (14 days)	6.87 ± 1.02^#^	7.65 ± 1.13^#^*	2.759	< 0.001
IL-4 (pg/mL)	Pre-treatment (1 h)	1.23 ± 0.21	1.27 ± 0.17	0.797	0.429
	Post-treatment (14 days)	3.82 ± 0.26^#^	3.21 ± 0.29^#^*	−8.434	< 0.001
IL-10 (pg/mL)	Pre-treatment (1 h)	2.29 ± 0.22	2.31 ± 0.17	0.387	0.700
	Post-treatment (14 days)	4.97 ± 0.24^#^	4.16 ± 0.25^#^*	−12.587	< 0.001

^#^*P* < 0.05 compared with pre-treatment within the same group.

*P < 0.05 compared with the study group at the same time point.

### 3.7 Correlation between clinical improvement and biomarker changes

**FIGURE 6:**
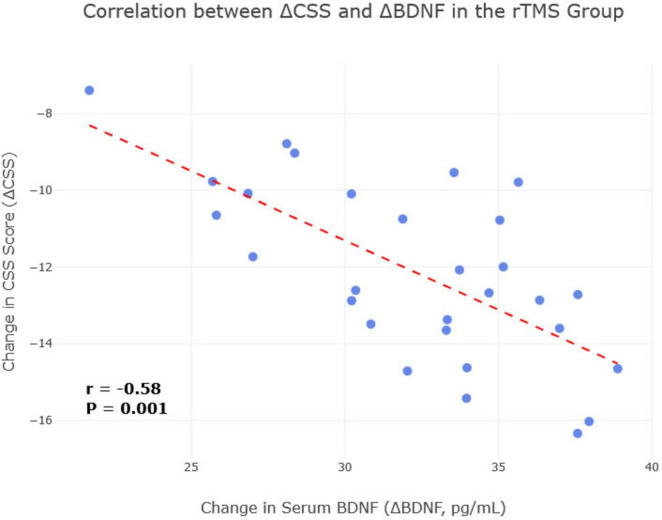
Correlation between change in brain-derived neurotrophic factor (BDNF) and change in Chronic Constipation Severity (CSS) score in the repetitive transcranial magnetic stimulation (rTMS) treatment group. A scatterplot visualizing the significant negative correlation between the change in CSS score and the change in serum BDNF levels in the rTMS treatment group (*n* = 29). Each point represents an individual patient. The line of best fit illustrates that a greater increase in BDNF (ΔBDNF) is associated with a greater reduction in the CSS score (ΔCSS) (r = –0.58, *P* = 0.001).

To further investigate the mechanistic link between biological and clinical changes, a Pearson correlation analysis was conducted in the rTMS treatment group (*n* = 29). A significant negative correlation was found between the change (improvement) in CSS scores (ΔCSS, calculated as post-treatment score minus pre-treatment score) and the change in serum BDNF levels (ΔBDNF, post-treatment minus pre-treatment) (r = −0.58, *P* = 0.001), indicating that greater symptom improvement was associated with a larger increase in BDNF (Figure 6). Similarly, a significant negative correlation was observed between ΔCSS and the change in serum IL-10 levels (ΔIL-10) (r = −0.62, *P* < 0.001), suggesting that a more robust anti-inflammatory response was linked to better clinical outcomes. No significant correlation was found between ΔCSS and changes in other biomarkers.

## 4 Discussion

Parkinson’s disease (PD) is a progressive neurodegenerative disorder where non-motor symptoms like constipation significantly impair quality of life (Leta et al., 2022). The pathophysiology of PD-related constipation is complex, involving central neurodegeneration, α-synucleinopathy in the ENS, and dysregulation of the gut-brain axis (Kulcsarova et al., 2023; Moin et al., 2022). Standard treatments like lactulose often yield suboptimal results (Kuai et al., 2021; Pedrosa Carrasco et al., 2018). This study investigated the efficacy of rTMS over the left DLPFC as an adjunctive therapy for constipation in PD patients and explored its impact on relevant gut-brain peptides and cytokines.

Our findings demonstrate that 14 days of active rTMS, compared to sham stimulation, led to significant improvements in clinical measures of constipation. Specifically, the rTMS group showed greater reductions in CSS scores, and greater increases in SBM, CSBM frequencies, and BSS scores, with effect sizes ranging from medium to very large. Moreover, a significantly higher percentage of patients in the rTMS group achieved a clinically meaningful reduction in constipation severity. These clinical benefits align with previous research suggesting rTMS can alleviate non-motor symptoms in PD (Cui et al., 2024; Liu et al., 2024) and modulate gut function (Li et al., 2023). For instance, Xu et al. (2022), Li et al. (2023) reported positive effects of neuromodulatory interventions on constipation in PD and functional bowel disorders by targeting the gut-brain axis. The remarkably consistent improvement in CSBM frequency in the study group (indicated by a small SD) further underscores the potential reliability of rTMS effects in a responsive population, though this observation merits cautious interpretation and verification in larger cohorts.

The mechanisms underlying rTMS effects on constipation likely involve multifaceted modulation of the gut-brain axis. We observed significant changes in serum gut-brain peptides. Post-treatment, the rTMS group exhibited higher levels of 5-HT and BDNF, and lower levels of VIP compared to the control group. Serotonin (5-HT) is a critical neurotransmitter in the ENS, primarily involved in stimulating gut motility; increased 5-HT could thus enhance colonic transit (Claudino Dos Santos et al., 2023). The rTMS-induced BDNF elevation correlated with constipation improvement (r = −0.58), suggesting enhanced neuroplasticity in gut-brain pathways may underlie symptom relief (Emmi et al., 2023; Palanisamy et al., 2022). Vasoactive intestinal peptide (VIP) typically inhibits gastrointestinal smooth muscle contraction; reduced VIP levels, as seen in our rTMS group, could disinhibit gut motility, thereby alleviating constipation (Palanisamy et al., 2022). Crucially, our correlational analysis strengthens this link, showing that greater increases in BDNF were directly associated with greater clinical improvement in constipation symptoms within the rTMS group. This aligns with studies showing that rTMS can upregulate BDNF, which may promote neuroplasticity within the central and enteric nervous systems (Li et al., 2020; Wang et al., 2011; Zheng and Tang, 2015). We hypothesize that rTMS of the DLPFC, a region involved in autonomic regulation, may modulate vagal outflow or neuroendocrine pathways, subsequently influencing ENS activity and peptide release (Napadow et al., 2008). However, as our study relied on serum markers, the causal link between these peptide changes and altered intestinal function remains inferential. Future research incorporating direct assessments of vagal nerve function (e.g., heart rate variability analysis) or neuroimaging techniques (e.g., fMRI) to visualize rTMS-modulated brain-gut connectivity would provide more direct evidence for these pathways.

Furthermore, rTMS appeared to favorably modulate the systemic inflammatory environment. Neuroinflammation is increasingly recognized in PD pathogenesis and can affect both central and enteric nervous systems (Eser et al., 2024; Samim Khan et al., 2023). Our study found that active rTMS led to significantly lower levels of pro-inflammatory cytokines (IL-6, IFN-γ, TNF-α) and higher levels of anti-inflammatory cytokines (IL-4, IL-10) compared to sham treatment, with effect sizes indicating these changes were of medium to very large magnitude. This anti-inflammatory shift, especially the significant association between increased IL-10 and symptom improvement, could be mediated by rTMS effects on the hypothalamic-pituitary-adrenal (HPA) axis or direct immunomodulatory actions (Xie et al., 2024). The full correlation matrix (Supplementary Figure 1) provides a comprehensive overview of these interrelationships, showing a consistent pattern where pro-inflammatory markers positively correlate with constipation severity, while anti-inflammatory and neurotrophic factors show the opposite trend. Reduced systemic and potentially enteric inflammation could contribute to improved gut barrier function and motility. Previous studies, such as by Xie et al. (2024), Hangzhou Normal University (2020), have also indicated that rTMS can mitigate inflammatory responses and improve clinical symptoms in PD, including constipation.

The discussion of mechanisms can be structured hierarchically. Firstly, direct neural modulation via rTMS influencing central autonomic control centers (e.g., DLPFC impacting vagal tone) could directly affect gut motility. Secondly, this central modulation can lead to altered secretion of gut-brain peptides (5-HT, BDNF, VIP) which act locally in the gut and also provide feedback to the brain. Thirdly, rTMS may exert immunomodulatory effects, reducing pro-inflammatory cytokines and increasing anti-inflammatory ones, which could alleviate neuroinflammation in both the brain and the gut, thereby improving ENS function. These pathways are not mutually exclusive and likely interact.

This study has several limitations. Firstly, the sample size (*n* = 58) is relatively small, which may limit statistical power and generalizability; future multi-center studies are warranted to confirm these findings. Secondly, the 14 days intervention period and lack of long-term follow-up data prevent assessment of the durability of rTMS effects. Thirdly, we did not analyze gut microbiota or microbial metabolites, a key component of the gut-brain axis. The causal inference between observed serum peptide changes and intestinal function is indirect, and direct measures of vagal activity or brain-gut connectivity were not performed. Furthermore, while our analysis did not formally adjust for potential confounders such as variations in concurrent PD mediations, we have now demonstrated that key baseline characteristics, including PD duration and initial constipation severity, were well-matched between the groups. While our statistical approach was appropriate for the study design, future research could benefit from more sophisticated models like repeated-measures ANOVA or mixed-effects models to better account for within-subject correlations over time. The determination of MT was based on visual observation, and while standard, EMG confirmation could offer more precision. The effectiveness of the sham stimulation in maintaining blinding was based on established methods but not formally assessed with a blinding index. While we instructed patients to maintain stable PD medication dosages, dietary habits, and physical activity levels, rigorous monitoring of these factors was not implemented, leaving potential for unmeasured confounding.

## 5 Conclusion

In summary, rTMS applied to the left DLPFC for 14 days significantly improved clinical symptoms of constipation in PD patients when compared to sham stimulation. These improvements were associated with favorable changes in serum levels of 5-HT, BDNF, VIP, and a shift toward an anti-inflammatory cytokine profile. Furthermore, the degree of clinical improvement correlated with the magnitude of increase in key biomarkers like BDNF and IL-10. These findings suggest that rTMS may exert its therapeutic effects by modulating the gut-brain axis through interacting neurochemical and immunological pathways. While promising, further research with larger cohorts, longer follow-up, and more direct mechanistic assessments is needed to substantiate these findings and establish the long-term clinical utility of rTMS for PD-related constipation.

## Data Availability

The original contributions presented in this study are included in this article/Supplementary material, further inquiries can be directed to the corresponding author.

## Supplementary material

Supplementary Figure 1 Correlation matrix of the changes in clinical scores and serum biomarkers in the repetitive transcranial magnetic stimulation (rTMS) treatment group (*n* = 29).
