# Supplementary material for: Clinical mechanisms of repetitive transcranial magnetic stimulation in improving constipation in Parkinson’s disease patients through the gut-brain axis
Source: Front Aging Neurosci. 2025 Aug 5;17:1607791. doi: 10.3389/fnagi.2025.1607791 (PMC12361172; doi:10.3389/fnagi.2025.1607791)
Supplement: Supplementary file 1 [file Table_1.docx]

**Supplementary Table 1: Results of Repeated-Measures ANOVA for Key Outcomes**

| **Variable** | **Effect** | **F(1, 56)** | **P-value** | **Partial Eta Squared (η²p)** |
| --- | --- | --- | --- | --- |
| **CSS Score** | Time | 894.62 | <0.001 | 0.941 |
|  | Time × Group Interaction | 67.46 | <0.001 | 0.546 |
|  | Group (Between-Subjects) | 8.54 | 0.005 | 0.132 |
| **CSBM Frequency** | Time | 2890.15 | <0.001 | 0.981 |
|  | Time × Group Interaction | 1450.59 | <0.001 | 0.963 |
|  | Group (Between-Subjects) | 18.11 | <0.001 | 0.244 |
| **BDNF (pg/mL)** | Time | 732.14 | <0.001 | 0.929 |
|  | Time × Group Interaction | 13.21 | 0.001 | 0.191 |
|  | Group (Between-Subjects) | 2.67 | 0.108 | 0.046 |
| **IL-10 (pg/mL)** | Time | 3578.91 | <0.001 | 0.985 |
|  | Time × Group Interaction | 158.44 | <0.001 | 0.739 |
|  | Group (Between-Subjects) | 14.32 | <0.001 | 0.204 |
| **IL-6 (pg/mL)** | Time | 2140.23 | <0.001 | 0.975 |
|  | Time × Group Interaction | 41.07 | <0.001 | 0.423 |
|  | Group (Between-Subjects) | 11.29 | 0.001 | 0.168 |

*Note: ANOVA was conducted on pre- and post-treatment values. The "Time × Group Interaction" is the primary outcome of interest, indicating whether the change over time differed significantly between the rTMS group and the control group.*
