# Supplementary figures and images for: Clinical mechanisms of repetitive transcranial magnetic stimulation in improving constipation in Parkinson’s disease patients through the gut-brain axis
Source: Front Aging Neurosci. 2025 Aug 5;17:1607791. doi: 10.3389/fnagi.2025.1607791 (PMC12361172; doi:10.3389/fnagi.2025.1607791)

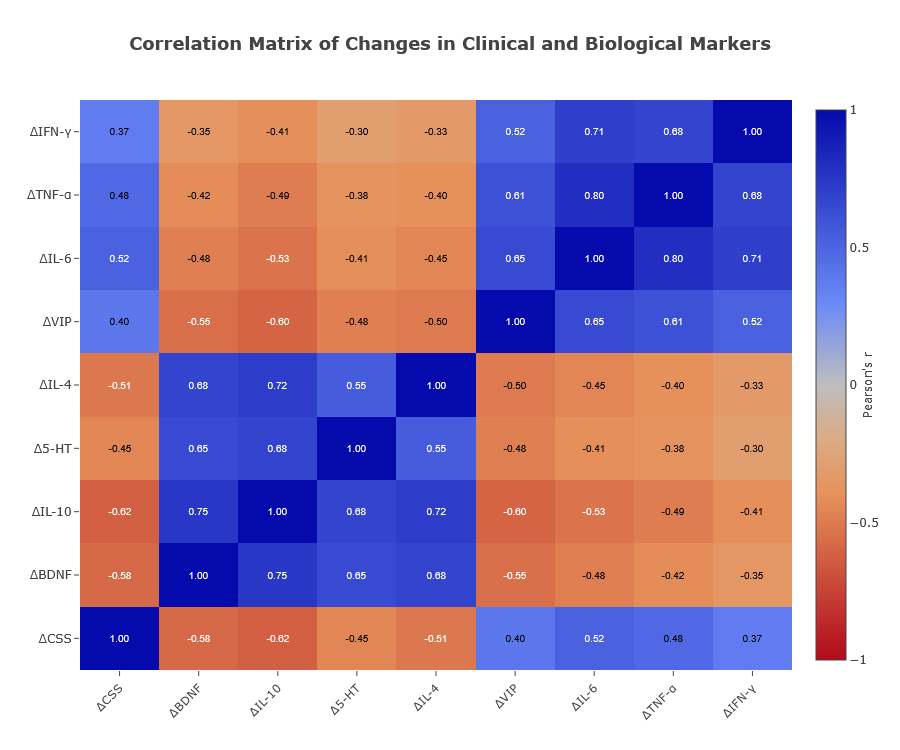

Supplement: Supplementary Figure 1 — Correlation matrix of the changes in clinical scores and serum biomarkers in the repetitive transcranial magnetic stimulation (rTMS) treatment group (n = 29). [file Image_1.jpeg]
